# Characterization of immortalized choroid plexus epithelial cell lines for studies of transport processes across the blood-cerebrospinal fluid barrier

**DOI:** 10.1186/1743-8454-7-11

**Published:** 2010-08-12

**Authors:** Juliane Kläs, Hartwig Wolburg, Tetsuya Terasaki, Gert Fricker, Valeska Reichel

**Affiliations:** 1 Ruprecht-Karls University, Department of Pharmaceutical Technology, 69120 Heidelberg, Germany; 2 Tübingen University Hospital, Institute of Pathology, 07071 Tübingen, Germany; 3 Tohoku University, Department of Biochemical Pharmacology and Therapeutics, Sendai 980-8578, Japan

## Abstract

**Background:**

Two rodent choroid plexus (CP) epithelial cell lines, Z310 and TR-CSFB, were compared with primary rat CP epithelial cells and intact CP tissue with respect to transport protein expression, function and tight junction (TJ) formation.

**Methods:**

For expression profiles of transporters and TJ proteins, qPCR and western blot analysis were used. Uptake assays were performed to study the functional activity of transporters and TJ formation was measured by trans-epithelial electrical resistance (TEER) and visualized by electron microscopy.

**Results:**

The expression of known ATP-binding cassette (Abc) transporter and solute carrier (Slc) genes in CP was confirmed by qPCR. Primary cells and cell lines showed similar, but overall lower expression of Abc transporters and absent Slc expression when compared to intact tissue. Consistent with this Mrp1, Mrp4 and P-gp protein levels were higher in intact CP compared to cell lines. Functionality of P-gp and Mrp1 was confirmed by Calcein-AM and CMFDA uptake assays and studies using [^3^H]bis-POM-PMEA as a substrate indicated Mrp4 function. Cell lines showed low or absent TJ protein expression. After treatment of cell lines with corticosteroids, RNA expression of *claudin1, 2 *and *11 *and *occludin *was elevated, as well as claudin1 and occludin protein expression. TJ formation was further investigated by freeze-fracture electron microscopy and only rarely observed. Increases in TJ particles with steroid treatment were not accompanied by an increase in transepithelial electrical resistance (TEER).

**Conclusion:**

Taken together, immortalized cell lines may be a tool to study transport processes mediated by P-gp, Mrp1 or Mrp4, but overall expression of transport proteins and TJ formation do not reflect the situation in intact CP tissue.

## Background

The blood-brain barrier (BBB) and the blood-cerebrospinal fluid (CSF) barrier (BCSFB) protect the brain from xenobiotics or other harmful molecules and ensure homeostasis of brain fluids. The BCSFB is formed by epithelial cells of the choroid plexuses (CP), which are located in the lateral, third and fourth ventricles [1]. Besides its barrier function, the CP produces and secretes CSF and supplies the brain with nutrients [2]. The paracellular route is obstructed due to tight junctions (TJs) between epithelial cells. Substance exchange between blood and CSF is mediated by several transport proteins, which actively take up certain substrates into epithelial cells and efflux substances out of the cells. CP amounts to only 0.25% of the brain [1] due to its small size (especially in rodents) and *in vitro *studies using primary rat CP epithelial cells are time and cost consuming. Therefore, CP cell line models have become important tools to study different pathological conditions and functions of CP. Two immortalized choroid plexus cell lines, TR-CSFB [3] and Z310 [4], have been developed from rat primary epithelial cells by transfection with SV40 large T antigen and are characterized in this study.

The ATP-binding cassette (Abc) and solute carrier (Slc) family play an essential role in transportation of a broad variety of substances and xenobiotics out of the brain, by uptake from CSF into epithelial cells and subsequent efflux into blood. Certain transporters of those families are known to be expressed in CP epithelial cells. Efflux transporters of the *Abcc *(multidrug resistance-associated protein, Mrp) family are expressed in CP tissue including Mrp1 [5], Mrp4 [6,7] and Mrp5 [7,8], while expression of P-glycoprotein (P-gp, *Abcb1*) may be more controversial. Rao *et al*. described P-gp protein expression in rat, mouse and human CP membrane fractions and sub-apical localization in rat CP, whereas Choudhuri *et al*. found P-gp RNA hardly to be expressed by the branched DNA assay method [5,7].

Mrp1 substrates include MK571 and indomethacin [9]. Mrp4 and Mrp5 show different substrate specificities than other Mrps, due to different structural characteristics. Substrates of those transporters include cyclic nucleotides such as cAMP and cGMP and nucleoside analogs like 9-(2-phosphonylmethoxyethyl)-adenine) (PMEA, adefovir) [10,11]. Mrp4 is inhibited by dipyridamole, dehydroepiandrosterone sulphate (DHEAS), MK571, sulindac sulfide, methotrexate and taurocholate (TC) [12,13]. P-gp is specifically inhibited by PSC833 (Valspodar^®^), LY335979 and GF120918 [14].

Organic anion transporters (Oats) belong to the *Slc22 *family. In rat CP epithelial cells *Slc22a7 *(Oat2) [7,15,16] and *Slc22a8 *(Oat3) expression was described previously [15-17].

Organic anion transporting polypeptides (Oatps) are members of the *Slco/Slc21 *family and *Slco1a4 *(Oatp1a4) was found to be expressed in rat CP and located on the basolateral membrane of the epithelial cells [18]. *Slco1a5 *(Oatp1a5) is abundantly expressed and located at the brush border membrane [19].

Hitherto, the two immortalized rat CP cell lines TR-CSFB and Z310 were characterized according to expression of the typical choroid plexus epithelial cell marker transthyretin (TTR) [4]. Expression of Na^+^/K^+^-ATPase, Mrp1 and Oatp1a5 has been described in TR-CSFB cells [20]. Functional activity could be demonstrated for Oatp1a5 by estrone-3-sulfate efflux [21]. In the Z310 cell line, expression of TTR receptor TfR and the transport proteins P-gp, Mrp1, Oat3 and organic cation transporter (Oct1, *Slc22a1*) have been described [4].

TJs connect CP epithelial cells and restrict solute movement through the paracellular pathway. In rodent CP, TJ proteins claudin1, 2 and 11 and occludin have been verified [22]. Expression of claudin1 (0.04% of CP), 2, 4, 8 and occludin (70% of CP) as well as its cytosolic binding partner, zonula occludens (ZO) 1-3, have been demonstrated in Z310 cells [23]. In TR-CSFB cells occludin has been found to be expressed at a lower level than in Z310, while claudin1 had higher expression at the protein level [24]. Trans-epithelial electrical resistance (TEER) in Z310 cells was enhanced by use of collagen-coated polyester filter membranes and treatment with glucocorticoids like dexamethasone. The sucrose permeability coefficient was also decreased [25].

The aim of the current study was to characterize TR-CSFB and Z310 CP cell lines in respect of the expression of Abc and Slc transport proteins and the functionality of the highly expressed transporters P-gp, Mrp1 and Mrp4. Furthermore, the expression of TJ proteins and TJ formation was investigated.

## Methods

### Chemicals

Cell culture devices and kits were purchased from the following companies: Dulbecco's modified essential medium (DMEM), DMEM-Ham's F12, as well as fetal bovine serum (FBS), Penicillin/Streptomycin, and Kanamycin, L-glutamine from Biochrom (Berlin, Germany); RNeasy kit, qPCR primer and QuantiFast SYBR green PCR kit from QIAGEN (Hilden, Germany), Complete™EDTA-free; Protease Inhibitor Cocktail Tablets from Roche (Mannheim, Germany), iScript™RT PCP kit from Biorad (München, Germany) Antibodies: anti-ABCC1 MAb [MRPr1], anti-p-glycoprotein MAb (C219) were ordered from Alexis Biochemicals (Lörrach, Germany) and anti-ABCC4 MAb [M4I-80] was obtained from Abcam (Dresden, Germany), mouse anti-Occludin and mouse anti-Claudin1 from Invitrogen (Karlsruhe, Germany). Secondary antibodies: Horseradish peroxidase conjugated anti-mouse and horseradish peroxidase conjugated anti-rat were purchased from Abcam, Western Lightning^® ^PLUS ECL, Enhanced Chemiluminescence Substrate, Ultima Gold™, Perkin Elmer (Rodgau, Germany), 25% Glutaraldehyde were obtained from Serva (Heidelberg, Germany), [^3^H]bis-POM-PMEA from Hartmann Analytics (Braunschweig, Germany), CellTracker™Green CMFDA was purchased from Invitrogen (Karlsruhe, Germany) and PSC833 was a kind gift from Novartis Pharma (Basel, Switzerland). All other chemicals were purchased from Sigma-Aldrich (Steinheim, Germany).

### Choroid plexus isolation

All experiments with animals were performed in accordance with German legal authorities and central animal facility of the University of Heidelberg (TV51/09). Male Wistar rats (230- 250 g) were cervical dislocated after isofluoran inhalation and the brains were removed and dissected in order to remove the CPs from lateral and third ventricles. CPs were either transferred into RNALater^® ^for RNA extraction or into CelLytic ™with protease inhibitor for protein isolation.

### Cell culture

Detailed information about TR-CSFB and Z310 culturing can be found in [3] and [4]. Briefly, TR-CSFB and Z310 cells were grown in DMEM media; TR-CSFB cells at 33°C and Z310 at 37°C with 95% humidity and 5% CO_2_. For TEER measurements 2x 10^5 ^cells/well were seeded on collagen-precoated transwell^® ^polyester membranes.

### Primary rat CP cell culture

For primary rat CP cell culture, 10-14 Wistar pups (P2) were used according to the protocol of Strazielle and Ghersi- Egea [26]. In brief, lateral CPs were dissected from each hemisphere and collected in pre-warmed culture medium (DMEM-Ham's F12, 10% FBS, 2 mM L-glutamine, 4 mM kanamycin and 10nM EGF). CPs were washed in PBS twice and incubated in PBS with 1mg/ml protease (from *Strepoyces griseus*) added for 25 min at 37°C while shaking at 300 rpm. Predigested CPs were allowed to sediment for 5 min and washed with PBS once. Then the following procedure was repeated three times: pellets were shaken in 1ml 0.025% trypsin (PBS) for 5 min at 37°C at 300 rpm, centrifuged for 5 min at 800 × g. Afterwards the pellet was re-suspended in 10 ml of pre-warmed culture medium, seeded to 25cm^2 ^flasks and incubated at 37°C, 95% humidity and 5% CO_2_. After 2 h non-attached cells were taken and seeded to collagen-coated transwell filters (for TEER measurement) or 25cm^2 ^flasks (for RNA isolation).

### RNA extraction and qPCR

RNA was extracted either from a 75cm^2 ^flask of 90% confluent cells or from freshly isolated choroid plexuses dissected from 5-8 Wistar rats by following the RNeasy protocol. RNA concentration was measured with NanoDrop. 1 μg of RNA was converted to cDNA by using iScript™cDNA synthesis kit. 50ng cDNA were used for qPCR. All primers were purchased from QIAGEN (QuantiTect Primer Assay: Abcc1: QT01811803, Abcc2: QT00374038, Abcc3: QT00191716, Abcc4: QT01804040, Abcc5: QT00179634, Abcc6:QT00188048, Slc22a6: QT00191261, Slc22a7: QT00177674, Slc22a8: QT00188937, Slco1a1: QT01084622, Slco1a4: QT00178815, Slco1a5: QT01829793, Slco2b1: QT00188489, Slco4a1: QT00193564, Pgp: QT00372673, Abcg2: QT00173138, Gapdh: QT00199633, Ocln: QT00196357, Cldn1: QT00399931, Cldn2: QT02356396, Cldn11: QT00176148) and tested with RNA extracted either from rat kidney or liver tissue (positive control) if not expressed in CP. The QuantiTect Primer Assays guarantee to be accurate and to be already optimized. http://www.qiagen.com/products/pcr/quantitect/primerassays.aspx#Tabs=t1. The following qPCR conditions were used: amplification 5 min at 95°C, 45 cycles of 10 sec 95°C, 30 sec 60°C and melting curve analysis 15 sec 95°C, 15 sec 65°C and final cooling to 40°C. Quantification cycle (Cq) values of each gene were normalized to the reference gene Gapdh (2^ΔCq^_GAPDH_^-ΔCq^_GOI_) in each group.

### Protein extraction

Membrane proteins were extracted according to protocol [27] with slight modifications. Cells were harvested from a 175cm^2 ^flask of 90% confluent cells, centrifuged and pellets resuspended in 35ml ice-cold hypotonic buffer (0.5 mM NaH_2_PO_4_, 0.5 mM Na_2 _PO_4_, 0.1 mM EDTA, pH7) with one tablet of Complete™EDTA free protease inhibitor cocktail. Then they were shaken for 45 min at 4°C and ultracentrifuged at 100,000 × g at 4°C. Pellets were resuspended in Tris-sucrose buffer (10 mM Tris-HEPES, 250 mM sucrose, pH7.4) with 1 tablet Complete™EDTA protease inhibitor and homogenized 30 times with a Dounce-homogenizer. To remove cell waste, centrifugation was performed for 15 min at 1,000 × g, before supernatant was used to gain membrane proteins by ultracentrifugation at 100,000 × g at 4°C for 1 h. Finally, the pellet was re-suspended in 500 μl Tris-sucrose buffer and protein concentrations were measured by Bradford protein assay [28]. Freshly isolated lateral CPs of 5 Wistar rats were used to extract whole protein following the CelLytic™protocol. In brief, CPs were collected in 1ml CelLytic™reagent containing 1:50 protease inhibitor on ice and stirred for 1 h. Centrifugation was performed for 10 min at 12,000 × g at 4°C; supernatant contained proteins.

### Western blot

Bio-Rad (München, Germany) system was used for immunoblot analysis. Proteins (10 μg) were diluted in 5x sample buffer (25%Gycerol, 7.5% SDS, 337 mM Tris, add freshly: 250 mM DTT, 0.25% bromphenol blue) and denatured at 95°C for 5 min. According to protein size 8-15% SDS gels were used; for Mrp1, 4 and P-gp: 10%, for claudin1: 15% and for occludin: 8%. Immunoblotting was performed for 1 h at 250mA and blocking for 30 min. Primary antibodies were added in an 1:100 (anti-Mrp1, anti-Mrp4, anti-P-gp) or 1:250 (anti-claudin1, anti-occludin, anti-Na^+^/K^+^-ATPase) dilution in blocking solution (5% milk) and incubated overnight at 4°C. Then 3x 10 min washing steps with PBST followed, before secondary antibodies (HRP conjugated anti-mouse for occludin, claudin1, P-gp and HRP conjugated anti-rat for Mrp1 and Mrp4) were added in a 1:5.000 dilution and incubated for 2 h at room temperature. Visualization of proteins was performed with Western Lightning^® ^PLUS ECL and bands were detected by QuantiOne Software with Molecular Imager ChemiDoc XRS System.

### Transport studies

For transport assays collagen-coated 96 well plates were seeded with a density of 2x10^4 ^cells per well and cultured for 4-5 days. Preliminary to the experiment, cells were washed with PBS twice. In uptake assays cells were pre-incubated with or without (control) inhibitors for 30 min at 37°C. All substances were dissolved in DMSO. Final DMSO concentration in incubation solutions was < 1%. Incubation with substrates and inhibitors was carried out for 90 min at 37°C. Subsequently, cells were washed with ice-cold PBS for at least three times to determinate transport and lysed with 1% Triton X100 in PBS for 1 h at 60°C. Analyses were performed either by fluorescent plate reader (Ascent Fluoroscan) or Scintillator (Packard Tri-Carb Liquid Scintillation Counter). Blank measurement was performed with cells treated only with PBS and 1% Triton X100.

### Trans-epithelial electrical resistance (TEER) measurement

To determinate the influence of hydrocortisone and dexamethasone on electrical resistance, cells were grown on collagen-precoated transwell^® ^polyester membranes and treated with hydrocortisone (100nM and 550nm) in serum-free medium for 4 days according to [29] or dexamethasone 1 μM for 8 days according to [25]. CellZscope^® ^(NanoAnalytics, Münster, Germany) was used for TEER measurement.

### Electron microscopy

Cells were seeded in flasks and treated with hydrocortisone and dexamethasone as described above. After 4 or 8 days in culture, cells were incubated with 2.5% glutaraldehyde for 4 h and washed with PBS twice. Freeze-fracturing was performed after fixation of the cells in 2.5% glutaraldehyde buffered with 0.1 M cacodylate buffer (pH 7.4) for 2 h at RT. Then the cells were scraped off from the substrate and impregnated with 30% glycerol in cacodylate buffer for 30 min, shock-frozen in nitrogen slush, transferred into a Balzers freeze-fracturing device BAF 400 D (Balzers, Liechtenstein), fractured at about 10^-6^mbar and -150°C, and shadowed with platinum/carbon (2.5nm, 45°) and carbon (25nm, 90°). The replicas were cleaned in 13% sodium hypochlorite, washed several times in distilled water, mounted on Pioloform-coated copper grids and observed in a Zeiss EM10 electron microscope (Zeiss, Oberkochen, Germany). Photographic negatives were digitized and images were arranged in Adobe Photoshop.

### Statistical analysis

Presented data are means ± SE with n ≥ 3. For statistical analyses of uptake assays unpaired student's t-test (Prism4.0 GraphPad software) was used (*P *values < 0.05 *, < 0.01 **, < 0.001***).

## Results

### RNA expression of transport proteins quantified by real time PCR

**Figure 1:**
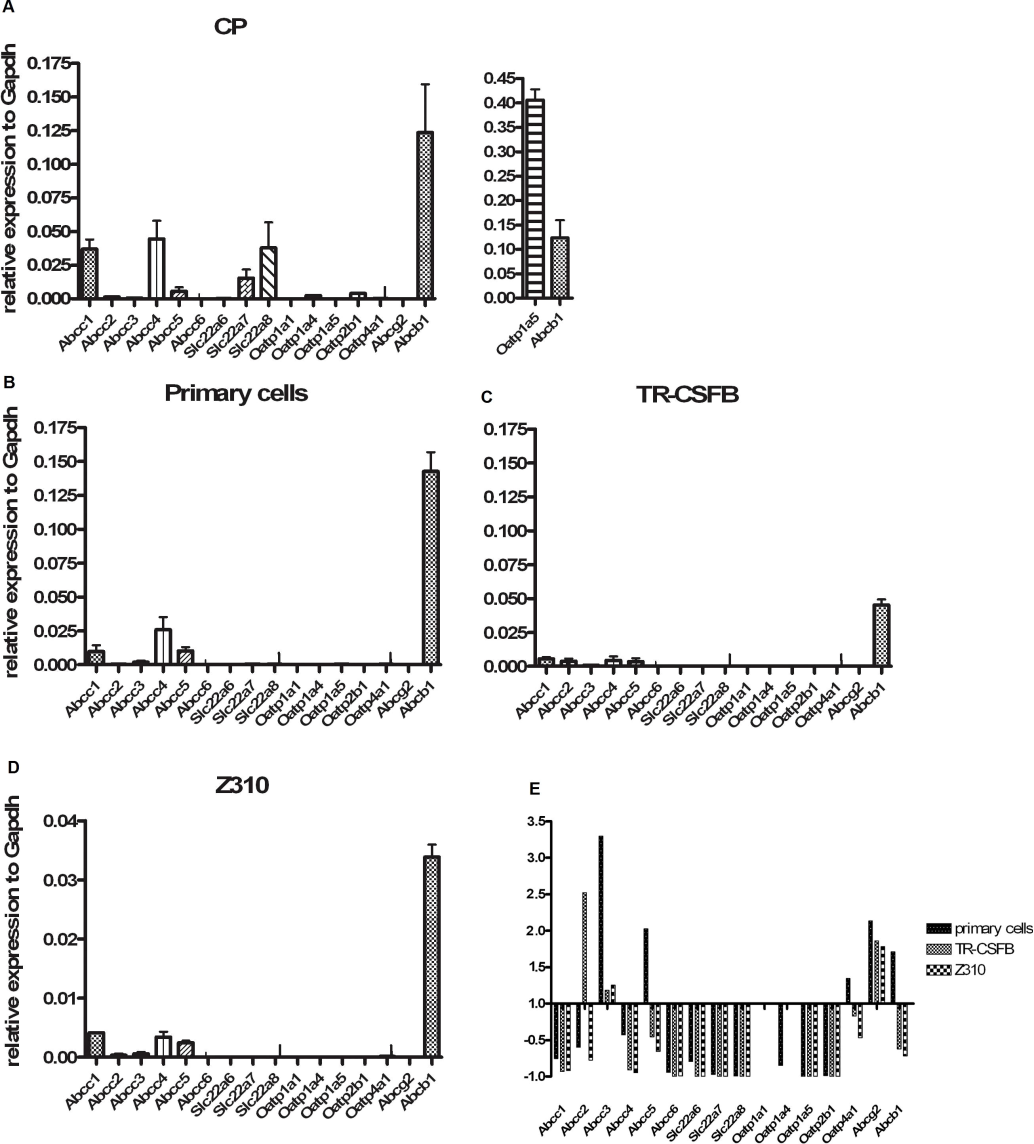
**A-D: Relative expression of the Abc and Slc transporters is shown as mean ± SEM in isolated CP, primary CP cells and in the immortalized cell lines TR-CSFB and Z310**. Cq (quantification cycle) values for each gene were normalized to Cq values of the reference gene Gapdh. *Slco1a5 *(Oatp1a5) is the most highly expressed gene in CP and shown in the separate graph. *Abcb1 *(P-gp), *Abcc1 *(Mrp1) and *Abcc4 *(Mrp4) are highly expressed in all cells as well as in the tissue control, while *Slc *genes are neither expressed in primary cells nor in the cell lines. E: Comparison of transporter expression in the cells with expression in CP tissue, which was set to 1. Most transporters are down-regulated in the cells. *Abcc2 *(Mrp2) is only expressed in TR-CSFB cells while primary cells express more *Abcc5 *(Mrp5) and *Abcb1 *(P-gp) than freshly isolated CP. *Abcc3 *(Mrp3) and *Abcg2 *(Bcrp), which seem to be up-regulated are hardly expressed as can be seen in B-D.

Comparison of transporter expression on RNA level in freshly isolated CP with primary rat CP epithelial cells and immortalized cell lines was performed by qPCR. We confirmed expression of transporters previously described in rat CP: *Abcc1, 4 *and *5 *(Mrp1, 4 and 5) as well as *Slc22a7, 8 *(Oat 2, 3) and *Slco 1a4, 1a5, 2b1 *and *Abcb1 *(P-gp) (fig. 1A). Primary cells as well as immortalized cell lines show a similar expression profile of Abc transporters with an overall lower expression than in CP tissue, but absent expression of Slc transporters (fig. 1B-D). Expression of *Abcc1, 4 *and *5 *as well as *Abcb1 *was slightly higher in primary cells compared to immortalized cell lines. In the TR-CSFB cell line *Abcc2 *(Mrp2) RNA was expressed, but could not be detected in rat CP, primary rat CP epithelial cells and Z310 cells. In contrast to a previous study [19]; *Slco1a5 *expression could not be detected in the TR-CSFB cell line.

Graphs in fig. 1A-D show the expression levels in CP and cells as values according to 2^ΔCq^_GAPDH_^-ΔCq^_GOI_. Fig. 1E displays an overview of the relative expression in cells compared to RNA expression in freshly isolated CP. Some transporters seem to be up-regulated in cells, including *Abcc3 *(Mrp3) and *Abcg2 *(Bcrp), but overall their expression is still hardly detectable (fig. 1B-D). *Abcc5 *(Mrp5) and *Abcb1 *(P-gp) are higher expressed in primary epithelial cells compared to freshly isolated CP.

### Expression of Mrp1, 4 and P-gp at the protein level

**Figure 2:**
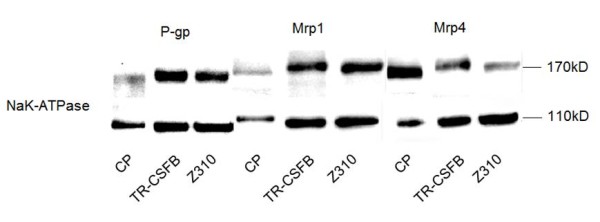
**Western blot analyses indicate expression of Mrp1, 4 and P-gp**. Whole protein was used from freshly isolated CP and membrane vesicles were isolated from cells (20 μg). There are slight differences in protein size possibly due to modifications in tissue and cells during processing.

Membrane proteins were isolated from the cell lines, while whole protein was used from rat CP, because, due to its small size, membrane isolation was not possible. CP tissue functioned as a positive control, Protein expression was not visualized in primary cells because we wanted to concentrate on characterization of the two immortalized cell lines. Abc transporters which showed expression at the RNA level in the cell lines were investigated for proteins expression. Mrp1, 4 and P-gp proteins were found in all three samples (fig. 2), and therefore further investigated for functional activity. Mrp5 could not be detected in the cell lines, only in freshly isolated CP (data not shown), therefore we did not consider Mrp5 to be relevant in the uptake studies of Mrp4. Mrp2 that was expressed at RNA level in TR-CSFB cells could not be detected at the protein level (data not shown).

### Uptake Assays

**Figure 3:**
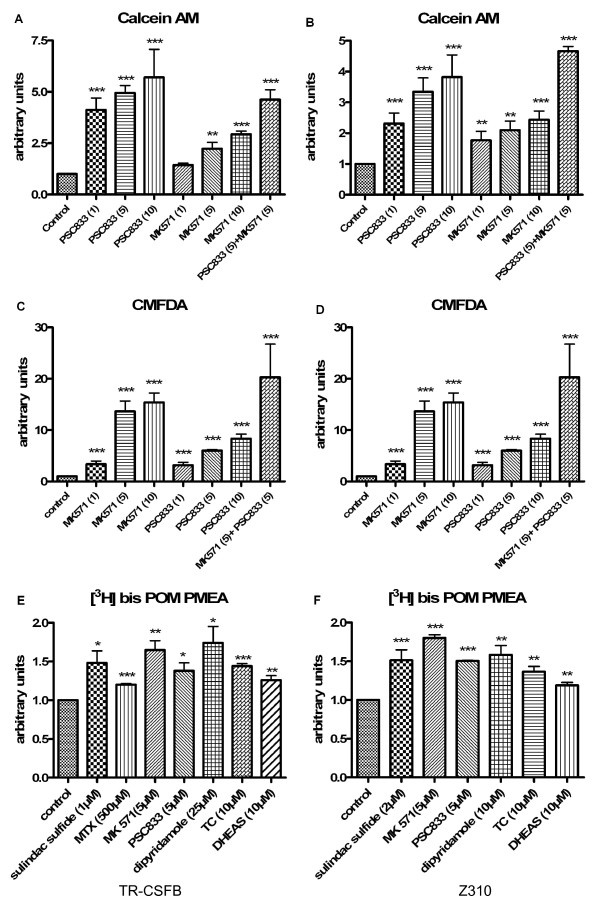
**A, B: Calcein-AM uptake assays show concentration-dependent inhibition by PSC833, MK571 and a cumulative effect of PSC833 and MK571**. Control was set to 1. C, D: CMFDA uptake assays show strong inhibition by MK571 and also moderate inhibition by PSC833. Again an additive effect by combination of PSC833 and MK571 is observed. * = significantly different from control, *P *< 0.05, ** = significantly different from control, *P *< 0.01, *** = significantly different from control, *P *< 0.001. E, F: Uptake of [^3^H]bis-POM-PMEA into TR-CSFB cells (E) and Z310 cells (F), respectively, was enhanced by sulindac sulfide, MK571, dipyridamole, TC (taurocholate), DHEAS (Dehydroepiandrosterone) and PSC833 acting as inhibitors of Mrp4 mediated efflux. A, C and E show TR-CSFB cells, B, D and F show Z310 cells. * = significantly different from control, *P *< 0.05, ** = significantly different from control, *P *< 0.01, *** = significantly different from control, *P *< 0.001.

In order to discover whether TR-CSFB or Z310 cell lines can be used for analysis of transport protein function, functional studies were performed using Calcein-AM, CellTracker™Green CMFDA and bis-POM-PMEA. Calcein AM diffuses inside the cell where intercellular esterases cleave the substance and fluorescent Calcein remains inside the cell or as a substrate of P-gp or of Mrp1, is effluxed by those transporters. Therefore, functional activity of P-gp and Mrp1 was confirmed by Calcein AM uptake assay in presence of the P-gp specific inhibitor PSC833 and Mrp specific inhibitor MK571 (fig. 3A, B, ** = significantly different from control, *P *< 0.01, *** = significantly different from control, *P *< 0.001). CellTracker™Green CMFDA (5-chloromethylfluorescein diacetate), a substrate of Mrp1 was used to further confirm Mrp1 functional activity. MK571 showed a strong inhibitory effect and concentration dependency. In addition, PSC833 showed an inhibitory effect on CMFDA efflux leading to elevated fluorescence levels inside the cells (fig. 3C, D, *** = significantly different from control, *P *< 0.001). A cumulative effect of MK571 and PSC833 could be demonstrated for both uptake assays, accumulation is stronger in CMFDA compared to Calcein AM assay (fig. 3A-D).

[^3^H]bis(pivaloyloxymethyl)-9-[2-(phosphonomethoxy)ethyl]-adenine (bis-POM-PMEA), an acyclic nucleoside phosphonate and substrate of Mrp4 was used to investigate Mrp4 activity. Uptake was increased in presence of sulindac sulfide, dipyridamole, TC (taurocholate), DHEAS (dehydroepiandrosterone), PSC833 and MK571 for both cell lines (fig. 3E, F, * = significantly different from control, *P *< 0.05, ** = significantly different from control, *P *< 0.01, *** = significantly different from control, *P *< 0.001), presumably by inhibition of Mrp4 mediated efflux. Further, bis-POM-PMEA was used as an inhibitor in Calcein AM and CMFDA assay and demonstrated no inhibitory influence on Mrp1 and P-gp, suggesting Mrp4 not to be involved in transport of these two substances (data not shown).

### Tight junction expression on RNA and protein level

**Figure 4:**
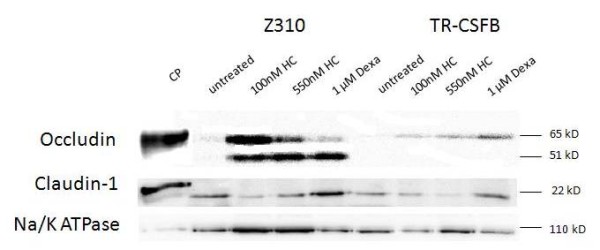
**Western blots with anti-occludin antibodies and anti-claudin-1 show higher expression of the two TJ proteins in Z310 compared to TR-CSFB cells and slightly increasing expression after treatment with glucocorticoids, dexamethasone (Dexa) and hydrocortisone (HC)**. 20 μg of each protein were applied. Occludin shows 2 bands, representing the phosphorylated momomeric subunit at 65kD and the unphosphorylated subunit at 51 kD. After detection of occludin and claudin-1, blots were stripped and Na/K-ATPase was detected.

Models of the BSCFB should reflect barrier properties and the formation of TJ is critical to achieve this. Untreated cell lines showed low or absent expression of the TJ proteins claudin1, 2, 11 and occludin (Table 1). In TR-CSFB cells, RNA expression of *claudin 1, 2 *and *occludin *was up-regulated after 8 days of treatment with 1 μM dexamethasone. *Occludin *expression was elevated after treating cells with hydrocortisone. Z310 cells treated with 550nM hydrocortisone displayed the highest RNA expression of *claudin 1, 2 *and *occludin*. Compared to freshly isolated rat CP, TJ RNA expression was 10-100 times lower in cell lines. Primary cells showed a somewhat higher expression, but were not comparable to freshly isolated tissue (table 1). To verify whether TJ proteins are expressed in the immortalized cell lines and expression may be up-regulated after dexamthasone or hydrocortisone treatment, western blot analyses were performed. At protein level TR-CSFB cells revealed higher expression of occludin after treatment with dexamethasone and hydrocortisone (fig. 4). In Z310 cells expression of occludin and claudin1 was up-regulated after hydrocortisone and dexamethasone treatment. Overall the increase in expression of claudin1 and occludin proteins was higher in Z310 than in TR-CSFB cells (fig. 4).

**Table 1:** RNA expression of TJ proteins occludin, claudin 1, 2 and 11

		TR-CSFB		Z310		primary cells		CP	
		
		**values shown as 2**^**ΔCq**^_**GAPDH **_**- **^**ΔCq**^_**GOI **_**× 10**^**3**^							
		
		Average	SE	Average	SE	Average	SE	Average	SE
claudin1	untreated	0.017	0.006	0.017	0.003	13.024	2.220	67.913	1.077
claudin1	100nM HC	0.346	0.070	2.521	0.121				
claudin1	550nM HC	0.478	0.053	**3.743**	0.422				
claudin1	1 μM Dex	**2.610**	0.124	1.424	0.135				

claudin2	untreated	1.907	0.699	0.203	0.090	3.566	0.329	364.891	59.225
claudin2	100nM HC	0.417	0.161	3.976	0.068				
claudin2	550nM HC	0.450	0.186	**6.635**	0.661				
claudin2	1 μM Dex	**4.686**	1.715	0.965	0.302				

claudin11	untreated	0.326	0.066	0.051	0.012	5.112	2.717	3.215	0.682
claudin11	100nM HC	0.042	0.014	0.103	0.019				
claudin11	550nM HC	0.067	0.030	0.119	0.025				
claudin11	1 μM Dex	**0.427**	0.280	0.054	0.009				

occludin	untreated	0.130	0.067	3.002	1.120	15.652	5.865	72.499	6.147
occludin	100nM HC	**6.580**	1.181	**34.882**	3.896				
occludin	550nM HC	**4.943**	1.237	**37.174**	4.639				
occludin	1 μM Dex	**7.899**	1.286	17.922	4.243				

mRNA expression of the CP TJ proteins *claudin1, 2, 11 *and *occludin *in TR-CSFB and Z310 cells comparing untreated cells with 100nM, 550nM hydrocortisone and 1 μM dexamethasone treated cells as well as with primary cells and CP tissue. Cq values were normalized to the reference gene GAPDH according to 2^ΔCq^_GAPDH_^-ΔCq^_GOI _and multiplied with 10^3 ^for better readable format (values in table). Bold values indicate high or elevated RNA expression. *Claudin1 *and *claudin2 *were highly up-regulated in TR-CSFB cells after treatment with dexamethasone, while hydrocortisone treatment had a higher impact on *claudin1 *and *2 *RNA expression in Z310 cells. *Occludin *on the other hand was up-regulated by all corticosteroid treatments, but again dexamethasone had a higher impact on its expression in TR-CSFB, while hydrocortisone elevated *occludin *expression in Z310 twice as much as dexamethasone.

### TEER measurement in TR-CSFB and Z310

**Figure 5:**
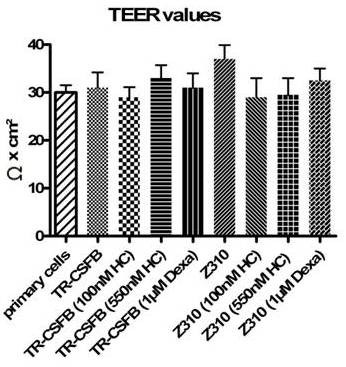
**Trans-endothelial electrical resistance (TEER) values of TR-CSFB and Z310 cells treated with 100nM and 550nM hydrocortisone measured in serum- free medium for 4 days or 1 μM dexamethasone for 8 days**. Untreated primary cells were used as control. Overall untreated and treated cells were grown on collagen-precoated transwell^® ^polyester membrane plates for 14 days.

Corticosteroid treated and untreated cells of both cell lines resulted in similar, low TEER values of 30-40 Ωcm^2 ^with no tendency of elevation after treatment. Primary cells also showed TEER values of about 30 Ωcm^2 ^(fig. 5).

### Visualization of TJs under electron microscope

In order to verify the actual formation of tight junctions rather than the expression of single proteins, cells were treated with hydrocortisone and dexamethasone and investigated by electron microscopical freeze-fracture analysis. TJ formation could not be verified by this method.

**Figure 6:**
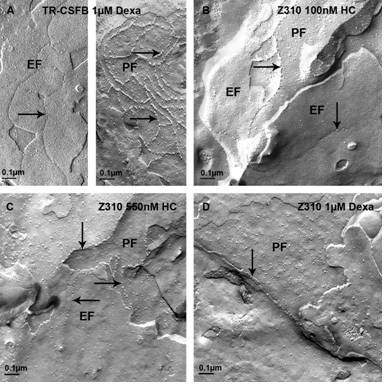
**Freeze-fracture replicas from TR-CSFB cells (A) and Z310 cells (B-D) treated with 1 μM dexamethsone (dexa. A, D) and 100 nM (B) and 550nM (C) hydrocortisone (HC)**. Whereas the TR-CSFB cells (A), but not the Z310 cells (D), have been partly induced by dexamethasone to form tight junctions (TJs), the Z310 cells (B,C), but not the TR-CSFB cells (not shown), can be induced by hydrocortisone to form some TJs. Arrows point to TJs particles in all pictures. EF: external fracture face, PF: protoplasmic fracture face.

Neither the untreated TR-CSFB nor Z310 cells showed any tight junctions in the freeze-fracture replica. In addition, the TR-CSFB cells did not react on hydrocortisone treatment with appearance of tight junctions (data not shown). However, dexamethasone treatment was answered by rare but consistent formation of tight junction networks associated predominantly with the P-face (fig. 6A). This result is consistent with the up-regulation of tight junction proteins in this cell line by dexamethasone (table 1, fig. 4). In contrast, and again in accordance with the biochemical data, dexamethasone did not increase the number of tight junctions in Z310 cells (fig. 6D), but in these cells tight junctions were slightly induced by hydrocortisone (fig. 6B, C). However, these tight junctions never formed continuous strands or networks, but insulated strands which probably would not increase the TEER. Indeed, this assumption was confirmed by the TEER values remaining constantly low after treatment with hydrocortisone or dexamethasone (fig. 5)

## Discussion

In this study expression of the major known CP ABC transporters *Abcc1 *(Mrp1), *Abcc4 *(Mrp4) and *Abcc5 *(Mrp5) could be confirmed at the RNA level in freshly isolated tissue as well as in primary cell culture and the immortalized cell lines TR-CSFB and Z310. Compared with the RNA expression profile of CP transporters from Choudhuri *et al*. in 2003 [7], we found *Abcc *transporters to be expressed in a very similar ratio. *Abcc1 *and *Abcc4 *display a 4 to 5 time higher expression than *Abcc5 *in Choudhuri's studies, whereas our profile indicates a 7-8 times higher expression. *Slco1a5 *has been shown to be the most highly expressed gene in both studies, whereas *Slco1a4 *and *1b2 *seem to have very low expression. *Slc22a7 *and *8 *are both expressed, but in our profile *Slc22a7 *is about half of the expression of *Slc22a8 *(fig. 1A), in Choudhuri's profile it is 3% of *Slc22a8*. The major difference of the two profiles is the expression of P-gp, which is very high in our study and very low in the other. The differences in the two expression profiles might be due to strain differences or gender-dependent deviations. In our study we used only male Wistar rats (230-250g) while Choudhuri *et al*. used both genders of Sprague- Dawley rats (150-200 g). Also primer specificity and method variations might have influenced the outcomes. In our work, we used qPCR, while Choudhuri *et al*. performed the expression profiles with the branched DNA assay method, which is based on hybridization not replication and no RT-PCR is needed to produce cDNA.

The two cell lines show a very similar expression profile with minor differences like RNA expression of *Abcc2 *in TR-CSFB cells (fig. 1C). *Slc *transporters that are present in CP could be confirmed neither in primary rat CP epithelial cells nor in the cell line models. The loss of those transporters might be due to the cell isolation process or conditions in the growth media. It has been shown that immortalization with SV40 large T antigen can lead to an increase of cytokines like IL-1 [30], which was demonstrated to down-regulate expression of hepatic organic anion transporters [31]. Taking a closer look into the pathway regulation of Slc expression might reveal the reason. A controversial result concerning Oatp1a5 was published by Ohtsuki [21] who found *Slco1a5 *to have very low expression at the RNA level and could confirm its activity by esterone-3-sulfate transport. Since the same cells were used at lower passage numbers (P51-P54) compared to our experiments (P67-P73), there might be a loss of expression in this cell line over time [32].

The controversial Abc transporter P-gp has been shown to be present in rat CP before [5] and to be expressed but not functionally active in porcine CP [33]. Choudhuri *et al*. 2003 [7] on the other hand found very low *Abcb1 *RNA expression in rat CP. Our findings demonstrated expression in freshly isolated rat CP and cell lines at the RNA (fig. 1) and protein level (fig. 2). In addition Calcein AM assay in TR-CSFB and Z310 showed significant inhibition by the P-gp specific inhibitor PSC883 (fig. 1A, B). Calcein-AM assay also revealed inhibition by Mrp1 inhibitor MK571 (fig. 3A, B), which might confirm Mrp1 activity, because Calcein AM is also transported by Mrp1 [34]. CellTracker™Green CMFDA, a substrate of Mrp1 and 2 [35] was used to further confirm Mrp1 functionality. Since Mrp2 was not expressed at the protein level it could be neglected. MK571 showed a strong inhibitory effect in the CMFDA assay, PSC833 treatment also led to increased intracellular fluorescence, but to a lower extent (fig. 3C, D). A combination of both inhibitors led to cumulated effects, which leads to the assumption that both, P-gp and Mrp1, actively participate in transport of Calcein and CMFDA, respectively. The Mrp4 substrate [^3^H]bis-POM-PMEA (fig. 3E, F) diffuses into cells [11] and uptake was inhibited by Mrp4 inhibitors sulindac sulfide, dipyridamole, taurocholate, DHEAS and MK571 [12,36]. Interestingly PSC833 also inhibited the efflux of [^3^H]bis-POM-PMEA. It has been published that P-gp does not transport [^3^H]bis-POM-PMEA [37]. In order to confirm that bis-POM-PMEA has no influence on P-gp and Mrp1 transport, it was used as an inhibitor in Calcein AM and CMFDA assays, where it showed no influence up to 10 μM, pointing out that PSC833 indeed inhibits Mrp4 activity (data not shown). Taken together the results indicate that the inhibition by MK571, taurocholate, DHEAS, sulindac sulfide and dipyridamole is due to active efflux transport of [^3^H]bis-POM-PMEA via Mrp4.

### Tight junction expression and formation under the influence of corticosteroids

TEER values found in primary porcine CP epithelial cells revealed 100-150 Ωcm^2 ^[33] and in bull frog 170 Ωcm^2 ^[38]. In primary CP cells, TEER was 70-178 Ωcm^2 ^[4,26,39] and was found to be highest after 8 DIC. For Z310 cells, TEER values of 50-200 Ωcm^2 ^[4,25] were measured, while for TR-CSFB cells the value was about 50 Ωcm^2 ^[3]. Zheng *et al*. furthermore, confirmed lower [^14^C] sucrose permeability after treatment with 1 μM dexamethasone for Z310 cells, accompanied by up to 50% higher TEER values [25]. The influence of corticosteroids and serum-free medium on tightness of porcine CPEC monolayer was reported before [40], but could not be confirmed for the immortalized cell lines. We found TEER values of 30-40 Ωcm^2 ^for both cell lines as well as for primary cells using cellZscope^® ^measurement and no elevation of TEER values after treatment with hydrocortisone or dexamethasone in the cell lines (fig. 5). Our lower TEER values received for cell lines as well as for primary cells might be due to different measurement methods using cellZscope^® ^analyses in our study (for a detailed comparison of chopstick vs. cellZscope^® ^measurements, the reader can refer to http://www.nanoanalytics.com).

The outcome was further confirmed by electron microscopical freeze-fracture analysis, where TJs could be found only rarely after hydrocortisone and dexamethasone treatment (fig. 6). However, in no instance did tight junctions induced by the treatments (fig. 6), show an increase the TEER (fig. 5). Z310 cells revealed an overall higher expression profile than TR-CSFB cells which goes along with the lower TEER values (50 Ωcm^2^) that were published for TR-CSFB cells [3], but we could not detect any differences between TR-CSFB and Z310 cells in TEER nor in the appearance of tight junctions.

As stated above the elevated tight junction protein expression did not lead to the actual formation of TJs. Since other proteins like Junctional Adhesion Molecules (JAMs), Crumb (Crb) and a number of cytoplasmic peripheral proteins are also involved in the pathway of TJ formation the whole pathway would need to be investigated to find the reason why TJ are not formed in the cell models. Transfection with SV40 large T antigen might have an influence on the pathway [41].

## Conclusion

Our results lead to the suggestion that the immortalized cell lines have to be used carefully and transport data have to be interpreted cautiously, as the expression of transport proteins does not exactly reflect the *in vivo *situation. Nevertheless, these cell lines might be particularly helpful to study transport processes at the blood-cerebrospinal fluid barrier mediated by Mrp1, Mrp4 or P-gp.

## Competing interests

The authors declare that they have no competing interests.

## Authors' contributions

JK carried out all experiments with exception of electron microscopy. HW performed the electron microscopical freeze-fracture analysis. TT designed the TR-CSFB cells and provided us with important information. GF was involved in the intellectual concept and editing the manuscript. VR designed the experiments and interpreted the data. All authors have read and approved the final version of the manuscript.
